# Methodology to Determine Cause of Death for Stillbirths and Neonatal Deaths Using Automated Case Reports and a Cause-of-Death Panel

**DOI:** 10.1093/cid/ciab811

**Published:** 2021-12-15

**Authors:** Kay S Hwang, Lindsay Parlberg, Anna Aceituno, Janet L Moore, Shivaprasad S Goudar, Shiyam Sunder Tikmani, Sarah Saleem, Gowdar Guruprasad, Amit Revankar, Zaheer Habib, Sangappa M Dhaded, S Yogesh Kumar, Chaitali Raghoji, Varun Kusugur, Sneharoopa Pujar, Sana Roujani, Elizabeth M McClure, Robert L Goldenberg

**Affiliations:** 1 RTI International, Research Triangle Park, North Carolina, USA; 2 JN Medical College, KAHER, Belagavi, India; 3 Aga Khan University, Karachi, Pakistan; 4 JJM Medical College, Davanagere, India; 5 Columbia University, New York, New York, USA

**Keywords:** cause-of-death determination, data management, minimally invasive tissue sampling (MITS), neonatal mortality, stillbirths

## Abstract

**Background:**

Review of data from multiple sources is often necessary to determine cause of death for stillbirths and neonatal deaths, especially in low- to middle-income countries (LMICs) where available data may vary. The minimally invasive tissue sampling (MITS) procedure provides granular histologic and microbiologic data that clinical reports and verbal autopsies cannot provide. Expert panel evaluation of data from individual deaths can be resource-intensive but remains essential to accurately infer causes of death.

**Methods:**

The Project to Understand and Research Preterms and Stillbirths in South Asia (PURPOSe) study uses review panels to evaluate causes of death in 2 LMICs. To make the process manageable, a subset of the study variables was selected with professional input and organized into case reports. Case reports include clinical information, laboratory results, fetal or neonatal organ histology and polymerase chain reaction results from tissue obtained by MITS. Panelists evaluated the complete case report forms and then determined the cause of death based on available data.

**Results:**

Computerized case reports averaged 2 to 3 pages. Approximately 6 to 8 cases were reviewed and discussed per 1-hour panel meeting. All panelists were provided the same information; missing data were noted. This limited bias between panelists and across meetings. Study teams notably took ownership of data quality.

**Conclusions:**

Standardized case reports for cause-of-death determination panel evaluation improve the efficiency of the review process, clarify available information, and limit bias across panelists, time, and location.

Of all stillbirths and neonatal deaths worldwide, it is estimated that 98%–99% occur in low- to middle-income countries (LMICs) [1, 2]. Based on a review of available clinical information, a determination by a panel of experts is considered the preferred method to determine the cause of death [3]. However, panel reviews historically have been a time-consuming process when the entire medical record must be evaluated.

Clinical and research assessments that are understood to inform cause-of-death (COD) determination can include obstetric history, clinical course for the mother, clinical status of the fetus, newborn intensive care unit (NICU) evaluation for admitted preterm births, placental pathology, laboratory reports, complete diagnostic autopsy (CDA) or minimally invasive tissue sampling (MITS), molecular or culture-based microbiological test results, and verbal autopsies. MITS is a post-mortem biopsy technique that is an acceptable alternative to the gold standard of CDA that can facilitate COD determination by providing materials for histology and microbiology evaluations. MITS has been shown to be of particular utility in LMICs where CDA is seldom performed [4, 5]. Furthermore, the available information to determine COD in these low-resource settings, most commonly clinical records and verbal autopsies, are often unreliable; clinical record review and verbal autopsy alone may actually mislead accurate COD classification [6–8]. By establishing an expert review panel, researchers can address these discrepancies and improve COD determination.

Review panels have been used for the COD determination in the US Stillbirth Collaborative Research Network study, Study of Illness in Preterms in Ethiopia, and in the Cause of Death Determination Using Minimally Invasive Autopsy (CaDMIA) study, which was extended under the name CaDMIA-Plus [3, 9, 10]. The Child Health and Mortality Prevention Surveillance Network (CHAMPS) also used Determination of Cause of Death (DeCoDe) panels [11]. In these examples, the review panel required substantial time and resources because the available information was denser and lacked summary data.

The Project to Understand and Research Preterms and Stillbirths in South Asia (PURPOSe) study is a large study funded by the Bill and Melinda Gates Foundation to determine the COD for stillbirths and preterm neonatal deaths that was conducted in India and Pakistan. In preparation to efficiently ascertain the COD for 1400 cases of which there were 700 stillbirths and 700 neonatal deaths, we built on the CHAMPS DeCoDe process, which included defined criteria for ascribing causality for CODs and a process for panelists to convene and evaluate cases [11]. In addition to tailoring the methodology to the population of stillbirths and preterm births, we developed a standardized case report summary for PURPOSe [11, 12].

Here, we present the methodology used to produce standardized case reports with a sufficient but minimal set of data from clinical records, as well as the research data, including the results from MITS and the molecular analysis of resulting clinical samples for pathogens and toxins using polymerase chain reaction (PCR) TaqMan array cards. We also present the processes established to organize the panel review of the reports to inform COD determination across 2 LMICs for the PURPOSe study.

## METHODS

### Developing Streamlined Standardized Case Reports

#### Data Selection

As a first step, a conceptual framework was developed to define the data likely necessary for each COD determination using the PURPOSe study data collection forms [1]. All variables were evaluated to see if they could potentially inform COD for stillbirths, preterm neonatal deaths, or both. Potential biases in the selection of information to present were restricted by consulting a multidisciplinary team that consisted of clinicians, pathologists, epidemiologists, and public health analysts. Prior to initiating the reviews, the study team convened for roughly 2 hours to determine which data from each study form would be necessary to inform COD. The data included in the reports were limited to those deemed essential with the understanding that review panelists could request additional information, if necessary, including photographs captured during the external physical examination. Programs were then developed to generate case reports by organizing the selected information, deriving helpful information to add to the reports, and addressing missing data.

#### Data Presentation

**Figure 1.**
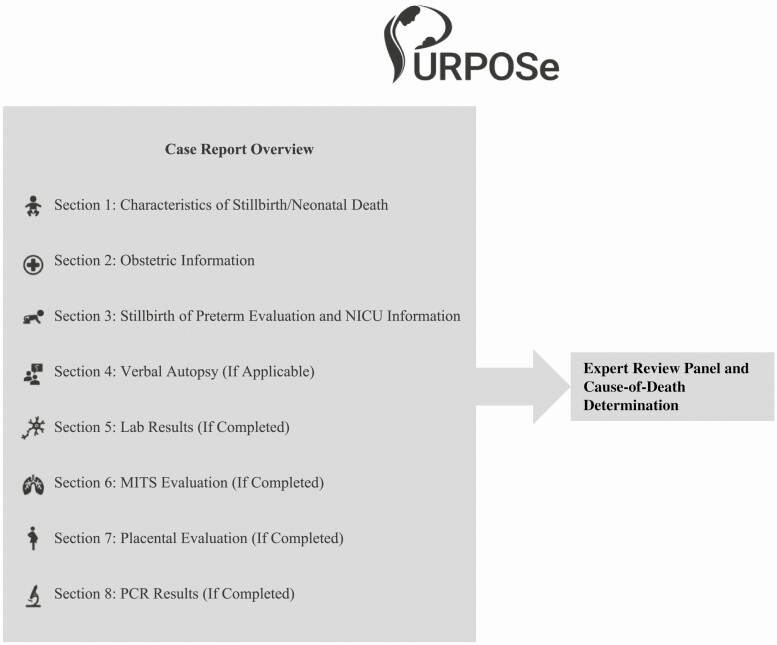
Case report overview. Abbreviations: MITS, minimally invasive tissue sampling; NICU, newborn intensive care unit; PCR, polymerase chain reaction.

To limit the quantity of information that would be presented within the targeted 2–3 pages per case, the report was organized in a consistent, easy-to-read layout and divided into 8 distinct sections (see Figure 1) starting with a summary of the death followed by a chronological overview of the chain of events related to each case.

The first section summarized basic birth characteristics such as birth weight, gestational age, and gender. The second section included obstetric data; the third section included the fetal or neonatal description, including pertinent conditions. An additional section was included for admitted preterm births and/or home deaths. The final sections included information from any completed research evaluations, including CDA (only for neonatal deaths in hospital from the India site), MITS, and laboratory findings.

In general, the format for displaying the clinical information was the characteristic followed by the case-specific data (eg, “birth weight (grams): 1800”). Certain groups of variables were combined into lists, by displaying all relevant conditions that were present or clinically abnormal. For example,

- “Positive tests: Rh factor, Active tuberculosis”
  - A negative Rh factor test signifies a clinically abnormal result, which triggers its appearance in the list for “positive” or clinically relevant tests.
- “Medical and obstetric disorders: Hypertensive disorders (Pre-eclampsia), Clinical chorioamnionitis”
  - Because hypertensive disorder is present, the type of disorder is followed in parentheses. Clarifying information from study subquestions is introduced only where applicable.

In many cases, the absence of a condition may be as informative as the presence of one. For example, group B *Streptococcus* (GBS) test results were shown regardless of result; a negative GBS test was determined to be a key important piece of data. Similarly, the absence of meconium at delivery was as informative as the presence of meconium. Other absent or “negative” test data were entirely omitted. For example, the PCR data provided results on 80 organisms for each sample tested, and each case had up to 8 samples. It was not helpful to list all organisms; therefore, we only listed the positive test results for each tissue sample. Clarifying how the data could inform COD was part of the initial review process after potentially informative variables were chosen.

#### Data-Driven Features

As previously mentioned, the preterm neonatal death cases included data from NICU visits that spanned the time from admission to final discharge. An infant may have multiple NICU visits with varying lengths of stay. Crucial data from each day in the NICU, including diagnoses, appearance, signs and symptoms, and treatments, were abstracted and presented. Summary data, such as total days in the NICU and age at death, were calculated and presented.

The reports also included reference information such as the International Fetal and Newborn Growth Consortium for the 21st Century 10th percentile birthweight based on gestational age and gender as well as the mean placental weight, which allowed the panel members to judge the case data in comparison to the published standards [13].

### Missing Data

It was critical to distinguish between the absence of a condition or characteristic and missing data. For all missing variables, “not answered” was displayed. Lists without any variables answered “yes” on the study forms showed “none” to indicate the lack of positive findings. The reduced information on the case report was organized by the study forms, and all variables were missing if an entire study form was not completed. In this case, only the section title that contained the name of the study form was printed along with the reason why (eg, if consent was not obtained or sample was not collected).

### Organizing the Expert Review Panels

Membership of the review panels consisted of obstetricians, neonatologists, pediatricians, and study staff, when clarification was needed.

Two members of the review panel performed the initial COD review for each case report. Their recommendation was considered the final COD unless there was a conflict or question. If so, the case in question was presented to the larger full panel for review. The final determination of COD used in the PURPOSe study was the responsibility of the review panel.

To standardize usage and improve quality of COD determination, all panel members attended an in-depth training workshop to ensure they understood how to interpret the findings presented on the standardized case report [14]. The workshop included a review of the format and data to be included in the reports with example scenarios and then practice cases. This process mirrored the CHAMPS DeCoDe training.

To effectively facilitate the expert review of 1400 cases, we established a comprehensive schedule of recurring panel pair conference calls. In advance of each call, the reviewers were expected to review the assigned reports and join the review call ready to determine COD for the assigned cases.

### Circulating Case Reports: Data Flow and Quality

**Figure 2.**
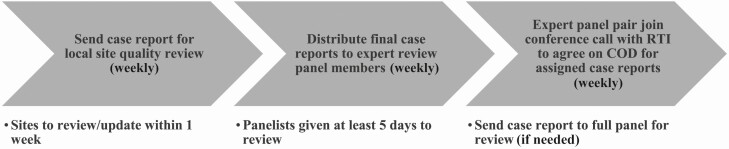
Case report data flow. Abbreviations: COD, cause of death; RTI, RTI International.

Case reports were sent to the data management teams at each local site for review of completion and accuracy before finalization for the panelists. The dates each report was sent to the sites and subsequently sent to the panelists were automatically tracked in a comprehensive Microsoft Excel workbook. This workbook detailed additional key case characteristics such as which study forms and analyses were completed to ensure that only cases with complete data were sent to panelists. Because the panelists thought it appropriate to review both infants of a multiple birth together, pregnancy fatalities of multiple pregnancies were reviewed together. Figure 2 describes the data flow.

### Assigning Cause of Death

The COD results were standardized as well. The PURPOSe study used the World Health Organization *International Classification of Diseases*, *Tenth Revision* (ICD-10), application to perinatal mortality (ICD-PM), as a basis for its coding [15]. In PURPOSe, however, we asked the panelists to determine a primary maternal cause, a primary placental cause, and a primary fetal or neonatal cause, assuming one was present. Multiple contributing causes for each of these primary causes could be assigned to ensure that these data were not lost and to avoid forcing the panelists to choose between various causes. For example, where the mother had preeclampsia, the placenta had vascular malperfusion lesions, and the fetus had an asphyxia episode, all of this important information could be captured as primary causes without forcing the panelists to choose among them. In addition, panelists could assign a level of certainty based on the available evidence to support the cause (ie, laboratory-based findings vs clinical symptoms). Finally, panelists noted whether the death could have theoretically been prevented, had evidence-based preventions, or treatments been applied.

## RESULTS

While it took approximately 2 days to initially abstract potentially useful data for COD determination and an additional month to create and refine the program to systematically abstract the data, the resulting consolidated, standardized case report summaries likely allowed for even more time saved. Using such summary reports, an average of 6 to 8 cases were reviewed and discussed per 1-hour meeting with panelists. For the PURPOSe study that has a total of approximately 1400 stillbirths and neonatal deaths, all eligible cases could be reviewed in about 200 panel hours, a manageable amount of time over the period of the project.

Panelists rarely requested other data or after discussion disagreed on the final determination, suggesting that the selected information on the summaries was sufficient. Throughout the review processes, we received positive feedback from our experts. They particularly appreciated the consistent format of each case report, which contributed to the ease of completing their review. Panelists had guidance on the information to expect in the cases for assigning COD, which aided in the review process and likely increased productivity over time as they became increasingly familiar with the organization of the report. Missing data, which can be challenging to verify with larger amounts of data, were made transparent by removing blank and unrelated information that may be typical on raw clinical forms from the case reports.

Information was prospectively selected for the case report based on its potential to inform COD without regard for the nature of the study. Some variables in the reports were not helpful in determining COD because they were rarely completed or clinically positive. For example, only 9 infants were screened for karyotype and zero infants were screened for any metabolic disorders. In retrospect, those variables could have been excluded, but they would have been relevant had the tests been ordered.

Furthermore, the addition of objective references for birth weight for gestational age and mean placenta weight limited possible bias from different medical trainings and cultural perceptions of mass and height across the 2 countries. Pulling together these references with clinical and pathological data in a digestible report facilitated the process of assigning COD by highlighting the complementary nature of the various sources of information. For example, a case may have a histologically normal placenta, but its small weight could suggest maternal vascular malperfusion.

Certain selected variables were found to be more helpful in assigning COD than others. Panelists considered the results of the MITS evaluation to be instrumental, especially in cases with limited clinical data. For example, panelists could confirm a case of respiratory distress syndrome (hyaline membrane disease) with the histological evaluation of the lung biopsy or could confirm suspected intrauterine hypoxia (asphyxia) if aspirated amniotic fluid or meconium was present in the lung biopsy, indicating the fetus was gasping in utero. Panelists noted that the placenta evaluation was particularly helpful in assigning COD for stillbirths.

An indirect benefit was the enhanced collaboration between diverse professionals (eg, pathologists, neonatologists) by providing digestible content to facilitate discussion. The cases were perceived as products of each site’s data, and the local study teams were encouraged to take ownership and responsibility of their data quality.

## DISCUSSION

Expert panel evaluation is the preferred method for COD determination, but it can be subjective and time-consuming [16]. The variation in available medical records, especially in LMICs, can make that process challenging. Developing a standardized case report that is specific to stillbirths and neonatal deaths and that systematically summarizes limited but sufficient and relevant information in a reproducible manner for the panelists to review in a timely manner, seems to address these concerns. All variables were first evaluated for the likelihood of contributing to the COD determination. The chosen information from various sources of data was then concisely organized. For completed cases, a draft report was sent to sites for initial quality review. Necessary changes to the data were made, and then the case report was finalized for the panelists’ review.

The case reports for the PURPOSe study had several unique features that helped improve efficiency of the panel review: most reports were only 2–3 pages; the formatting and order of information was consistent; missing data were clearly labeled; and despite the shortened length, the reports combined clinical, histologic, and microbiologic data. These elements allowed adequate time for panelists to review each report and were particularly important for a subjective task like determining COD. Furthermore, site staff were motivated to produce quality case reports and carefully reviewed the data and, if possible, addressed missing data.

The PURPOSe data entry process accelerated the consolidation of the multiple types of information we had available for each case. Data managers at each site and their teams completed the PURPOSe study forms, which required pulling information from different locations (eg, clinical records and microbiology results likely come from separate parts of the hospital). Other studies that use raw data forms and do not have a document management system in place would especially benefit from standardization of variables, though it may require greater initial effort to create the program to systematically produce case reports.

Developing a standardized format for organizing and reviewing clinical and research data appears to make the panel’s COD evaluation more efficient and, importantly, allows each of the panelists to have the same data at their disposal when cases are reviewed. The improvement is clear when comparing the COD review process between PURPOSe, CHAMPS, and CaDMIA. CHAMPS provided all forms to the panelists, which could total more than 90 pages and required considerable time to review. PURPOSe altered this process by abstracting relevant information and presenting it in 2–3 pages. Moreover, these summaries included not only the clinical chain of events for each infant death but also the histology and microbiology results in the same format, unlike the CaDMIA study that condensed clinical information and required references to additional databases for laboratory results. We acknowledge that the short length of the PURPOSe case reports was, in part, due to the limited data and short medical history of stillbirths and neonates, and that case reports for children, adults, and those with complicated medical histories would have more data and be inherently longer. However, the same data consolidation and streamlining process can be applied to these cases and may be even more helpful to clinicians who review these case reports in an objective and efficient manner.

While the reports offer standardization in the presentation of data, it is equally important to standardize the interpretation of the information. This classification system in place, based on ICD-PM, with the addition of placental causes, can reduce errors and improve accuracy of determining causes of stillbirths and neonatal deaths.

Understanding the causes for the high rates of stillbirths and neonatal deaths across LMICs can lead to interventions to reduce these deaths. Similar methods for case report creation, review, and interpretation when applied to future COD studies will increase efficiency of COD attribution, especially if paired with an evaluation of the usefulness of each type of information in determining COD. Standardization should improve comparability of resulting data.

## Notes

***Acknowledgments.*** We thank the Project to Understand and Research Preterms and Stillbirths in South Asia (PURPOSe) study teams in India and Pakistan for their continuous support and dedication, the expert panelists for the considerable time they provided for the benefit of this study, and the Child Health and Mortality Prevention Surveillance Network study for establishing the building blocks necessary for the determination of cause of death.

***Supplement sponsorship.*** This supplement is sponsored by MITS Surveillance Alliance Secretariat, led by RTI International, with funding from the Bill and Melinda Gates Foundation.

***Financial support.*** This work was supported by the PURPOSe study, funded by the Bill and Melinda Gates Foundation (Opportunity/Contract OPP1169824). Under the grant conditions of the foundation, a Creative Commons Attribution 4.0 Generic License has already been assigned to the Author Accepted Manuscript version that might arise from this submission.

***Potential conflicts of interest.*** All authors: No reported conflicts of interest. All authors have submitted the ICMJE Form for Disclosure of Potential Conflicts of Interest. Conflicts that the editors consider relevant to the content of the manuscript have been disclosed.
